# Detection and quantification of antibody to SARS CoV 2 receptor binding domain provides enhanced sensitivity, specificity and utility

**DOI:** 10.1016/j.jviromet.2022.114475

**Published:** 2022-04

**Authors:** Carolina Rosadas, Maryam Khan, Eleanor Parker, Federica Marchesin, Ksenia Katsanovskaja, Macià Sureda-Vives, Natalia Fernandez, Paul Randell, Ruth Harvey, Alice Lilley, Benjamin H.L. Harris, Mohamed Zuhair, Michael Fertleman, Samreen Ijaz, Steve Dicks, Charlotte-Eve Short, Rachael Quinlan, Graham P. Taylor, Kai Hu, Paul McKay, Annachiara Rosa, Chloe Roustan, Mark Zuckerman, Kate El Bouzidi, Graham Cooke, Barnaby Flower, Maya Moshe, Paul Elliott, Alexandra J. Spencer, Teresa Lambe, Sarah C. Gilbert, Hugh Kingston, J. Kenneth Baillie, Peter J.M. Openshaw, Malcolm G. Semple, Peter Cherepanov, Myra O. McClure, Richard S. Tedder, ISARIC4C Investigators

**Affiliations:** a Department of Infectious Disease, Imperial College London, St Mary’s Campus, London, W2 1PG, UK; b Department of Infection and Immunity, Imperial College Healthcare NHS Trust, Charing Cross Hospital, London, W6 8RF, UK; c Worldwide Influenza Centre, Francis Crick Institute, London, NW1 1AT, UK; d The Wellington Hospital, Circus Road, St John’s Wood, London, NW8 6PD, UK; e Computational Biology and Integrative Genomics, Department of Oncology, University of Oxford, Oxford, OX3 7DQ, UK; f Blood Borne Virus Unit, National Infection Service, Colindale Public Health England, London, NW9 5EQ, UK; g Transfusion Microbiology, NHS Blood and Transplant, Lingard Avenue, London, NW9 5BG, UK; h Chromatin Structure and Mobile DNA Laboratory, The Francis Crick Institute, London, NW1 1AT, UK; i Structural Biology Science Technology Platform, Francis Crick Institute, London, NW1 1AT, UK; j Department of Virology, King’s College Hospital, London, SE5 9RS, UK; k Department of Epidemiology and Biostatistics, School of Public Health, Imperial College London, St Mary’s Campus, London, W2 1PG, UK; l Jenner Institute, University of Oxford, ORCRB, Oxford, OX3 7DQ, UK; m Roslin Institute, University of Edinburgh, Midlothian, EH25 9RG, UK; n National Heart and Lung Institute, Imperial College London, Chelsea, London, SW3 6LY, UK; o NIHR Health Protection Research Unit in Emerging and Zoonotic Infections, Institute of Infection, Veterinary and Ecological Sciences, Faculty of Health and Life Sciences, University of Liverpool, Liverpool, L69 7BE, UK; p Crick COVID19 Consortium, Francis Crick Institute, London, NW1 1AT, UK

**Keywords:** Sars-CoV-2, ELISA, Receptor binding domain, Antibodies

## Abstract

Accurate and sensitive detection of antibody to SARS-CoV-2 remains an essential component of the pandemic response. Measuring antibody that predicts neutralising activity and the vaccine response is an absolute requirement for laboratory-based confirmatory and reference activity.

The viral receptor binding domain (RBD) constitutes the prime target antigen for neutralising antibody. A double antigen binding assay (DABA), providing the most sensitive format has been exploited in a novel hybrid manner employing a solid-phase S1 preferentially presenting RBD, coupled with a labelled RBD conjugate, used in a two-step sequential assay for detection and measurement of antibody to RBD (anti-RBD).

This class and species neutral assay showed a specificity of 100 % on 825 pre COVID-19 samples and a potential sensitivity of 99.6 % on 276 recovery samples, predicting quantitatively the presence of neutralising antibody determined by pseudo-type neutralization and by plaque reduction. Anti-RBD is also measurable in ferrets immunised with ChadOx1 nCoV-19 vaccine and in humans immunised with both AstraZeneca and Pfizer vaccines. This assay detects anti-RBD at presentation with illness, demonstrates its elevation with disease severity, its sequel to asymptomatic infection and its persistence after the loss of antibody to the nucleoprotein (anti-NP). It also provides serological confirmation of prior infection and offers a secure measure for seroprevalence and studies of vaccine immunisation in human and animal populations.

The hybrid DABA also displays the attributes necessary for the detection and quantification of anti-RBD to be used in clinical practice. An absence of detectable anti-RBD by this assay predicates the need for passive immune prophylaxis in at-risk patients.

## Highlights

• Detects antibodies which prevent the virus receptor from attaching to cells.
• Predicts and measures virus-neutralising antibody.
• Very high specificity and sensitivity.
• Identifies risk persons who would benefit from immune plasma therapy.
• Species and class neutral.

## 1 Background

In the field of infectious diseases, detection of a pathogen is dependent upon its culture or detection of viral genome or antigens, processes that are the cornerstones of diagnosis. The corollary of using antibody to detect the host response to infection has been undervalued. However, early studies on SARS-CoV-2 (Zhao et al., 2020) showing the magnitude and the rapidity of the immune response to viral infection underscore the utility of serology.

Point-of-care tests (POCTs) for serology have been rapidly taken up in the UK but while offering remote sampling and testing, the performance of such lateral flow antibody tests (LFAT) is variable and may not meet the minimum criteria demanded by the Medicines and Healthcare products Regulatory Agency, (MHRA) with resulting concern over their wider application (Flower et al., 2020). Moreover, the inevitable absence of quality assurance for the procedure of home testing renders insecure the data generated from the widespread of adoption of POCTs.

Detection of the host response to a pathogen is impacted on by the nature of the antibody response, including antibody class and the range of available antigens. Furthermore, the format of the serological test employed has a profound effect, as was graphically encountered during the Ebola epidemic in West Africa (Tedder et al., 2018). Conventionally many immunoassays for the detection of antibody are based on an indirect format whereby antibody binding to the solid phase antigen is revealed by a labelled antibody to human immunoglobulins. Such assays are simple to manufacture but can be fraught with problems of specificity partly due to alteration of epitope profile expressed by the complex protein represented by the corona virus envelop spike when adsorbed to a solid matrix. Also such indirect assays do not readily allow the use of analytes other that serum or plasma. The use of labelled viral antigens in a variety of assay formats can allow the detection of antibody in both serum or plasma, and in non-blood/serum analytes, avoiding also the need for invasive sampling (Tedder et al., 2018). A labelled antigen-revealing agent can also provide an opportunity for quenching non-specific reactivity arising from cross-reacting antibody directed at related, but irrelevant pathogens, well exemplified in flavi-virus serology (Tedder et al., 2019).

In the UK, assays based upon the nucleoprotein (NP) antigen, including the Abbott and the Roche platforms, detect antibody to NP (anti-NP) as evidence of prior infection. However, these fail to confirm *per se* the presence of antibody likely to confer immunity, albeit of unknown duration, against reinfection (Rydyznski Moderbacher et al., 2020). For this reason, we have explored the use of external components of the virus in the knowledge that an antibody response to the receptor binding domain (RBD) is likely to be predictive of neutralising antibody. Furthermore, the detection and quantification of anti-RBD is essential for detecting and characterising vaccine responses. Anti-RBD may also offer protection over and above that offered by the cytotoxic T-lymphocyte cellular response resulting from the primary infection (Azkur et al., 2020) and hyperimmune preparations of anti-RBD to be administered to anti-RBD seronegative patients considered “at risk” are increasingly available.

Three formats, not including the indirect format, were employed during the Ebola epidemic in West Africa (Tedder et al., 2018) for the detection of antibody to envelope components of the Ebola virus. We elected to reiterate this approach for SARS-CoV-2 using a double antigen binding assay (DABA) format. We describe here the construction, format and performance of a solid-phase sequential two incubation-step enzyme-linked immunoassay (ELISA) for the detection and quantification of anti-RBD). Uniquely we have employed an S1 protein constructed to retain RBD antigenicity in the face of biliverdin (Lumley et al., 2020) which preferentially expresses RBD when bound to the solid phase. We have uniquely paired this with labelled RBD in a hybrid DABA. We compare the performance of the Abbott assay for antibody to the nucleoprotein (anti-NP) to that of anti-RBD detected in the hybrid assay for the detection of prior SARS-CoV-2 infection. We demonstrate anti-RBD predicts a neutralising response and allows the class-neutral detection of antibody in individuals with past asymptomatic infection and in vaccine recipients, both humans and animals. We further confirm an enhanced anti-RBD response in relation to symptomatology and, importantly, confirm the durability of the anti-RBD antibody response in the recovery period.

## 2 Methods

### 2.1 SARS-CoV-2 antigens

The SARS-CoV-2 Hybrid DABA uses both the viral S1 and RBD antigens in a two-step double antigen binding format. The solid phase is coated with S1 antigen, while RBD conjugated with horseradish peroxidase (HRP) reveals captured antibodies. Both proteins were initially produced at The Francis Crick Institute, London. Subsequently, the S1 expression vector has been engineered to produce a protein that is antigenically stable in the face of biliverdin (Rosa et al., 2021). Both this protein and RBD are now expressed by, and now are sourced directly from The Native Antigen Company in Oxford both as native antigens, and where required as horseradish peroxidase-labelled antigens.

SARS-CoV-2 RBD conjugation was initially undertaken using the LYNX Rapid HRP Conjugation kit (Bio-Rad Laboratories Ltd, Watford, UK) according to manufacturer`s instructions. The conjugated RBD was diluted 1:10 in HRP Stabilising Buffer (ClinTech, Guildford, UK) and stored at −20 °C. Prior to use it was diluted to a final and determined optimum concentration in conjugate diluent (ClinTech, Guildford, UK).

### 2.2 Hybrid DABA immunoassay

Solid phase 96-microwells plates (NUNC Immunomodule, U8 Maxisorp wells) were coated with 100 μl of S1 antigen at a concentration of 5 μg/mL (MicroImmune Coating Buffer; ClinTech, Guildford, UK) overnight at 2−8 °C, followed by 3 h at 35−37 °C (under moist conditions) and 1 h at room temperature. Plates were washed once with 0.05 % Tween 20/PBS, blocked with MicroImmune Blocking Solution (3−4 hours at 37 °C in a moist box), aspirated, dried overnight at 37 °C and stored dry at 4 °C in sealed pouches with desiccant. The assay was carried out by adding 50 μl of sample diluent (MicroImmune Sample Diluent; ClinTech, Guildford, UK) to each well, followed by addition of 50 μl of control and test sera to respective wells. Plates were incubated for 1 h at 37 °C then washed five times with wash buffer (ClinTech, Guildford, UK). One hundred microlitres of the RBD-HRP conjugate appropriately diluted in Conjugate buffer (Clintech, Guildford, UK) were added to the wells, incubated for 1 h at 37 °C, washed five times and 100 μl of TMB substrate added (ClinTech, Guildford, UK), incubated for 30 min at 37 °C, after which the reaction was stopped and read spectrometrically at 450−630 nm. The cut-off was established by adding an arbitrary 0.1 to the average of optical density (OD) obtained for three negative controls assayed in each run. The signal/cut-off value (binding ratio, BR) for each sample was determined by dividing the sample OD by the cut-off OD. A sample was considered positive if BR ≥ 1. United Kingdom Patent Application No. 2011047.4 for “SARS-CoV-2 antibody detection assay” has been filed.

### 2.3 Assay validation

To evaluate assay specificity 825 stored plasma and sera pre-dating the SARS-CoV-2 outbreak were used. All samples were anonymised prior to SARS-CoV-2 serology testing (Table 1A). To evaluate assay sensitivity a panel of sera was obtained from 276 patients with mild or moderate clinical SARS-CoV-2 infection at least 14 days after symptoms onset (Table 1B).

**Table 1A:** The panel of 825 pre-SARS-CoV-2 samples.

**Nature of sample**	**Number**	**Source**	**Site**
Sera	94	Blood donors	Scottish National Blood Transfusion Service
Sera	498	Airwave (Elliott et al., 2014)	Imperial College
Plasma	100	Antenatal∼	NWL Pathology Service
Plasma	133	HTLV infected	National Centre for Human Retrovirology

∼ CDRTB https://directory.biobankinguk.org/Profile/Biobank/GBR-1-305.

**Table 1B:** The Panel of 276 samples from patients with mild or moderate clinical SARS-CoV-2 infection at least 14 days after symptoms onset.

**Sample Number**	**Source**	**Site**
103	REACT 2 (Flower et al., 2020)	Imperial College
51	CDRTB∼	Imperial College
122	STOICS	King’s College Hospital

Infection confirmed by PCR or at least one other serological assay (SARS-CoV-2 Abbott IgG, Wantai Total Antibody or ‘in-house’ indirect tri-spike assay).

∼ CDRTB https://directory.biobankinguk.org/Profile/Biobank/GBR-1-305.

### 2.4 Detection of neutralising antibody

#### 2.4.1 Pseudotype neutralisation assay

Twenty-eight anti-RBD-reactive samples, randomly selected, were tested for neutralising activity by a pseudotype neutralisation assay using an HIV-pseudotyped luciferase-reporter based system as previously described (Rosa et al., 2021). The CoV S-pseudotyped viruses were produced by co-transfection of 293 T/17 cells with a HIV-1 *gag-pol* plasmid (pCMV-Δ8.91), a firefly luciferase reporter plasmid (pCSFLW) and a plasmid encoding pSARS-CoV2-S at a ratio of 1:1.5:1. Heat-inactivated sera were serially diluted and incubated with virus for 1 h. Serum-virus mixture was transferred into wells with pre-seeded Caco2 cells. After 48 h, cells were lysed, luciferase activity was measured by Bright-Glo Luciferase Assay System (Promega) and the IC_50_ neutralisation titre determined. The coefficient of correlation between Hybrid DABA BR and the IC_50_ was calculated.

#### 2.4.2 Plaque-reduction assay

Forty samples selected for discordance between anti-RBD and anti-NP reactivity and 10 concordant samples were tested for plaque reducing antibody. Confluent monolayers of Vero E6 cells in 96-well plates were incubated with 10–20 PFU of SARS CoV-2 (hCoV-19/England/02/2020, EPI_ISL_407073, kindly provided by Public Health England) and two-fold serial dilution of antibodies for 3 h at 37 °C, 5% CO_2_, in duplicate per condition. The inoculum was then removed, and cells overlaid with virus growth medium containing Avicel at a final concentration of 1.2 %. Cells were incubated at 37 °C in 5% CO_2_. At 24 h post-infection, cells were fixed in 4% paraformaldehyde, permeabilised with 0.2 % Triton-X-100/PBS and virus plaques visualised by immunostaining, as described previously for the neutralization of influenza viruses (Lin et al., 2015), but using a rabbit polyclonal anti-NSP8 antibody and anti-rabbit-HRP conjugate and detected by action of HRP on a tetra methyl benzidine (TMB) based substrate. Virus plaques were quantified and IC_50_ for sera were calculated using LabView software, as described previously (Elliott et al., 2014).

### 2.5 Hybrid DABA (anti-RBD) assay performance compared to that of the Abbott Architect SARS-CoV-2 IgG Assay (anti-NP)

Samples from 2205 patients or health care professionals with clinically suspected or diagnosed SARS-CoV-2 infection were tested as a convenience sample using the manufacturer’s instructions and referred for confirmatory testing on the Hybrid DABA as part of an NHS service development initiative. A sample was considered positive in the Abbott assay if it has a signal/control ratio of 1.4 and above. Operators were blinded to sample sero-status at time of testing. The concordance between tests was analysed and Kappa index calculated.

### 2.6 Relationship between clinical illness, the magnitude of the antibody response and its durability

The relationship with clinical illness of the anti-RBD response was determined in asymptomatic staff and patients whose samples were referred from the Wellington Hospital, enrolled in the COVIDITY study and compared with samples from patients with severe symptoms from the ISARIC4C study (Lin et al., 2015). To investigate further the longitudinal stability of anti-RBD 737 samples from 109 patients with asymptomatic infection or mild disease were collected over a period of up to 58 weeks following onset of symptoms or the time of diagnosis for asymptomatic patients. The magnitude of the antibody response was investigated through quantification of 345 sera from 242 patients hospitalised with SARS-CoV-2 infection enrolled in the ISARIC4C study.

### 2.7 Quantification of antibody reactivity

The first WHO International Standard for anti-SARS-CoV-2 immunoglobulin (NIBSC, 20/136) was utilised to quantify antibody titres. The standard, ascribed 1000 Binding Antibody Units (BAU)/mL, was serially diluted to extinction in normal human plasma and assayed in the DABA in replicates. Optical densities (OD) and WHO units were plotted to give maxima for quantification and end point sensitivity. Sera, appropriately diluted in NHP, could be ascribed anti-RBD in BAU/mL against this curve. Four Parameter Logistic Curve equation was employed in order to create a sigmoid fitting curve of BAU/mL against OD values. The parameter values were initially attributed considering the minimum and maximum OD values recorded by the spectrophotometer (SpectraMax M2, Molecular Devices). Subsequently, for each run, the ‘Solver’ Program was used to calculate the four parameter values that give the lowest sum value of the square difference between OD recorded experimentally and OD calculated with the four parameter logistic equation. Sera frequently required a starting dilution of 1:100, often needing a further 10 or 100-fold. To obviate the need for pre-dilution in general use, a reduced serum input volume of 5 μL was also employed on occasions (Tedder et al., 2018). All dilutions and input volumes were factored to ascribe reactivity correctly to the original samples.

### 2.8 Measurement of the serological response to vaccine immunisation

#### 2.8.1 Response in ferrets

Twenty three plasma samples from ChAdOx1 nCoV-19 vaccine-immunised animals and 16 from ChAdOx1 GFP (an irrelevant antigen) immunised animals were available for testing from the Nuffield Department of Medicine, University of Oxford. Ferrets either received a single intramuscular dose of vaccine (2.5 × 10 (Lambe et al., 2021) vp) or two doses of vaccine 28 days apart (Lambe et al., 2021). Samples at day 28 (n = 11), day 35 (n = 6) and day 42 (n = 6) were available from ChAdOx1 nCoV-19 immunised animals and day 28 (n = 8), day 35 (n = 4) and day 42 (n = 4) ChAdOx1 GFP immunised animals.

#### 2.8.2 Response in humans

Forty samples from 28 individuals that were previously determined to be sero-negative with no history of SARS-CoV-2 infection prior to immunisation, were assayed to determine anti-RBD reactivity. Samples were taken pre-immunisation and at a minimum of 14 days post second immunisation with either the Oxford/AstraZeneca AZD1222 or the Pfizer-BioNTech COVID-19 Vaccine.

### 2.9 Ethics

Airwave samples are held in the Airwave Tissue Bank (http://police-health.org.uk/airwave-tissue-bank) and used with permission from the Airwave Access Committee. Other pre−COVID-19 sera from patients with HTLV1 infection were donated, following written informed consent, to the Communicable Diseases Research Tissue Bank (CDRTB) of the Section of Virology. The use of these tissues was approved by the CDRTB Steering Committee in accordance with the responsibility delegated by the National Research Ethics Service (South Central Ethics Committee – C, NRES reference 15/SC/0089). Anonymised, redundant sera from antenatal patients attending Imperial College Healthcare NHS Trust (ICHNT) were donated by NWLP. Staff and patients of ICHNT and the Wellington Hospital diagnosed with SARS-CoV-2 infection were also donated to the CDRTB (COVIDITY) following written informed consent (NRES 20/SC/0226).

Identified patients hospitalised during the SARS−CoV-2 pandemic were recruited into the International Severe Acute Respiratory and Emerging Infection Consortium World Health Organization Clinical Characterisation Protocol UK (IRAS260007 and IRAS126600). Written informed consent was obtained from all patients. Ethical approval was given by the South Central–Oxford C Research Ethics Committee in England (reference: 13/SC/0149), Scotland A Research Ethics Committee (reference: 20/SS/0028) and World Health Organization Ethics Review Committee (RPC571 and RPC572 l; 25 April 2013). The ISARIC WHO CCP-UK study was registered at https://www.isrctn.com/ISRCTN66726260 and designated an Urgent Public Health Research Study by NIHR.

To provide reference confirmatory testing for serum samples following the introduction of anti-NP screening, the presence of anti-RBD was sought in agreement with the Virology service of North West London Pathology at Charing Cross Hospital, ICHNT as part of an agreed NHS Service Development bid. Data arising from this service development are presented here.

## 3 Results

### 3.1 Assay specificity and sensitivity

**Fig. 1:**
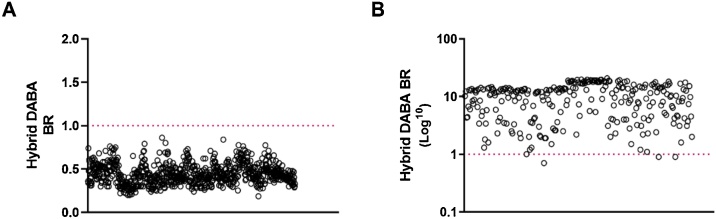
**Hybrid DABA specificity and sensitivity determination** Scatter plot distribution of the Hybrid DABA BRs of A) 825 archival serum samples that pre-dated the SARS-CoV-2 epidemic assayed and B) 276 samples from patients infected with SARS-CoV-2. Dotted line represents the assay cut-off. Test /Cut-off binding ratios (BR) are displayed on A) a linear scale and B) on a log_10_ scale.

All 825 archived pre−COVID-19 samples were unreactive (BR < 1) (Fig. 1A) indicating an assay specificity of 100 % (95 %CI = 99.6–100 %). Three out of 276 SARS-CoV-2 patient samples were unreactive in the Hybrid DABA, being two with a BR of 0.9 and one with a BR of 0.7, giving a sensitivity of 98.91 % (95 %CI = 96.8–99.8 %) (Fig. 1B). A ROC analysis using the MedCalc program (n = 276 seropositives; n = 825 seronegatives) indicated that with a BR of 0.86 the assay retained 100% specificity and achieved 99.6 % sensitivity.

### 3.2 Pseudotype neutralisation activity

**Fig. 2:**
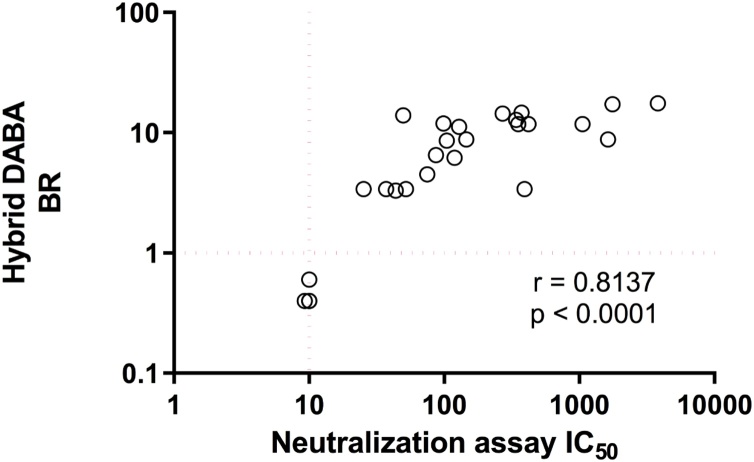
**Hybrid DABA BRs correlate with SARS-CoV-2 pseudo-type neutralising antibody titres.** The degree of correlation when 28 sera were assayed in the Hybrid DABA and the BRs compared to neutralising antibody titres, given as IC_50_ doses.

From a convenience panel of 28 DABA variously reactive samples, all 22 samples reactive in the DABA displayed neutralising antibodies. Six un-reactive samples had no detectable neutralising antibodies. The Hybrid DABA BR strongly correlated with SARS-CoV-2 Neutralisation IC_50_ (r = 0.81; p < 0.0001) (Fig. 2).

### 3.3 Comparison between hybrid DABA and Abbott IgG anti-SARS-CoV-2 assays

**Fig. 3:**
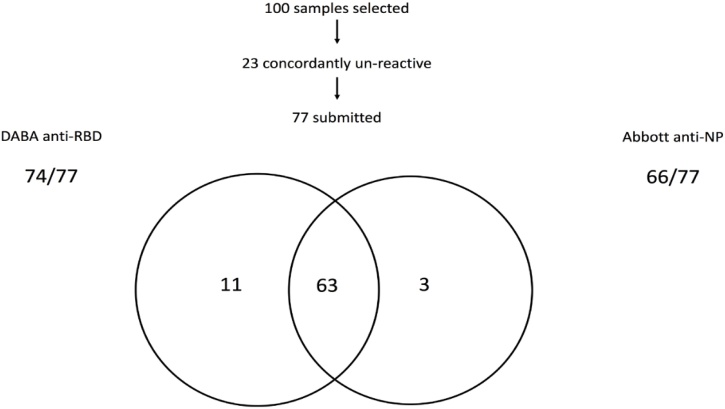
**Comparison of Hybrid DABA and Abbott Architect reactivity for antibody to SARS-CoV-2 in a panel of 100 samples**. The two-way Venn illustrates the outcome of 77 seropositive samples tested in both the Abbott assay (anti-NP) and the Hybrid DABA (anti-RBD). *Ten of the eleven were PCR-confirmed, the remaining ill patient was not tested by PCR. **One sample submitted for neutralising antibody was negative in the pseudo-type assay, DABA BRs were 0.9 in the other two.

The initial comparative analysis of 100 samples selected randomly from 100 individuals in the COVIDITY study showed that 23 of the 100 samples were concordantly negative by both assays and 63 concordantly positive **(**Fig. 3). Anti-NP testing failed to detect 11 samples from symptomatic persons reactive in the Hybrid DABA, 10 of which came from PCR-confirmed infections. The hybrid DABA did not detect three samples that were detected by Abbott, one of the three that was tested had no detectable neutralising antibodies by a pseudotype neutralisation assay. neutralising data were not available for the other two, both had BRs 0.9 in the Hybrid DABA, just below the cut-off.

**Fig. 4:**
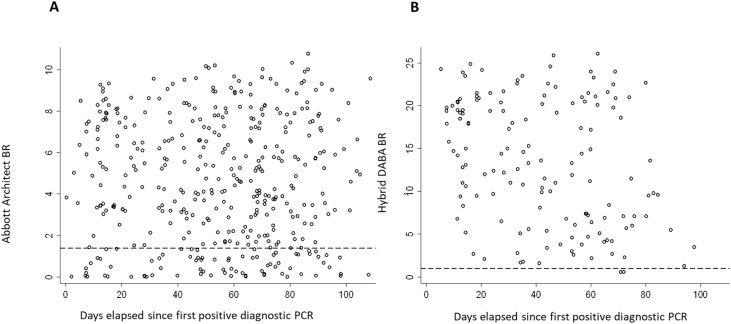
**Comparison of Abbott Architect and Hybrid DABA reactivity for antibody to SARS-CoV-2 in diagnostic samples referred for confirmatory anti-RBD testing.** Temporal analysis of unselected convenience samples tested initially for anti-NP prior to referral for further testing. The BRs displayed for the anti-NP Abbott (A) and for the anti-RBD hybrid DABA (B) with days elapsed from the first diagnostic PCR. Dashed lines represent the respective cut off BR values for each assay. Only samples with an Abbott BR > 0.25 were referred for anti-RBD screening.

Following the Abbott assay introduction in ICHNT a total of 2205 samples, were received for further testing for the presence of anti-RBD. Ranking reactivity to time from the first diagnostic PCR, indicated a better diagnostic accuracy for the hybrid DABA (Fig. 4).

Using the manufacturers’ criteria 511 samples did not contain detectable anti-NP on the Abbott assay, yet 294 (57.5 %) were reactive for anti-RBD (Table 2), as previously described in part (Rosadas et al., 2020).

**Table 2:** Reactivity for anti-RBD in 2205 samples previously submitted for antibody screening for anti-NP by Abbott Architect.

**No of Samples**	**Anti-NP Architect**	**Anti-RBD reactivity Hybrid DABA**	
	**BR range**	**Negative**	**Positive (%)**
196	0.25−0.5	129	67 (34)
101	0.51−0.75	41	60 (59.4)
95	0.76 to 1.0	28	67 (70.5)
70	1.1−1.25	16	54 (77.1)
49	1.26−1.39	3	46 (94)
906	1.4−2.5	72 (8)	834 (92)
787	>2.5	4 (0.5)	783 (99.5)
Totals : 2205		294 (13.3 %)	1911 (86.7 %)

Ranked by Architect binding ratios, the prevalence of detectable anti-RBD in the Hybrid DABA rises from 34 % with BRs 0.25 to 0.5, rising to 94 % in the 49 samples lying just below the manufacturer’s BR cut-off of 1.4.

**Fig. 5:**
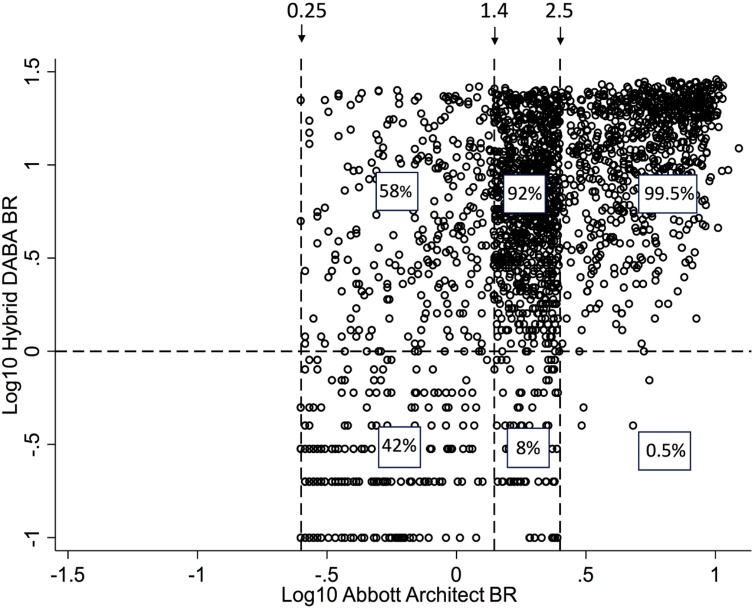
**BR Correlation between Hybrid DABA and Abbott SARS-CoV-2 IgG.** Correlation displays BRs expressed in Log_10_ values. Horizontal dashed line ids the BR for the hybrid DABA of 1.0. Vertical dasshed lines show the mininum Architect BR of 0.25 below which samples were not reffered for analysis, the manufacturers’ cutoff 1.4 and the upper range of 2.5. The correlation for the samples with strong eactivity ((Hybrid BR > 10 and Arcitect >2.5) is clearly seen.

Increasing reactivity for anti-NP at levels below the manufacturer’s cut-off predicted the presence of anti-RBD in the discordant samples. A significant correlation between DABA BR and the Abbott anti-NP BR **(**Fig. 5) was displayed by concordantly reactive samples (Kappa Index 0.654, 95 % CI 0.493−0.815) but was lost when comparing Abbott borderline samples when only a weak correlation was observed (r = 0.3292, p < 0.0001).

**Fig. 6:**
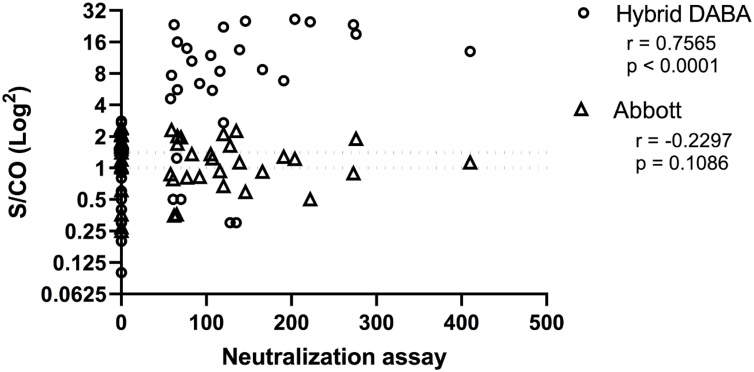
**Abbott and Hybrid BRs and their correlation with neutralising antibody titres.** Abbott assay borderline samples, their discordance when assayed in the Hybrid DABA and the correlation of the Hybrid results with neutralising antibody responses, determined by plaque-reduction assay. Dotted lines represent the cut off values (red DABA; blue Abbott).

Samples unreactive for anti-NP but reactive in the DABA frequently harboured detectable neutralising antibodies (85.7 %, 18/21), as measured by plaque-reduction assay. The majority of anti-NP positive borderline samples not containing detectable anti-RBD had no neutralising antibodies (82.4 %, 14/17). There was a strong correlation between anti-RBD BR and the titre of neutralising antibodies when testing borderline discordant samples (r = 0.7565, p < 0.0001) but no correlation between anti-NP BR and titres of neutralising antibodies (r=-0.2297; p = 0.1086) (Fig. 6).

### 3.4 Durability of the anti-RBD response

In most patients anti-RBD response could be detected in recovery. In a series of 737 specimens taken from 109 individuals, 68 samples from 18 patients were unreactive. Three of these patients had an initial negative sample early in illness and then seroconverted. Ten patients had initial positive samples but with low BRs in nine patients (BR 4.9 to 1.0) and then sero-reverted to become sero-negative (32 samples). One of the ten patients became sero-unreactive for anti-RBD as early as four weeks after onset of illness. This was a symptomatic patient (fever, cough, myalgia, headache and anosmia) with confirmed SARS-CoV-2 infection by RT-PCR. In the first sample (nine days after symptoms onset) the BR was 3.3 becoming borderline within one week and negative in subsequent follow-up samples.

**Fig. 7:**
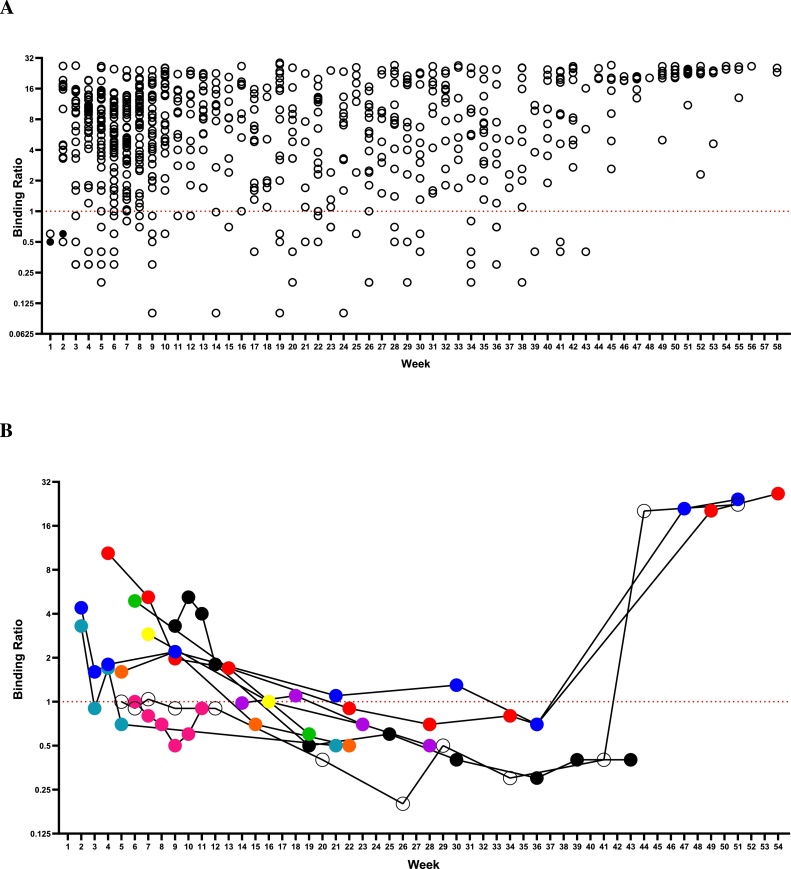
**Persistence of the anti-RBD antibody response**. A) Solid symbols represent those negative results from patients that that later seroconverted. BR displayed on log2 scale. Duration of follow-up shown in weeks. Dotted line is cut-off. A total of 737 samples are shown from 109 patients, 68 samples from 18 patients were unreactive. Three patients seroconvert during follow up. Ten sero-positive patients sero-revert to become sero-negative (32 samples). Five patients are persistently sero-negative (33 samples) but have a positive-PCR result listed, 2 of their samples displayed borderline binding ratios of between 0.8 and 0.9. B) Evolution of anti-RBD total antibody assayed over time in a minority of ten patients who sero-reverted during follow-up. Coloured symbols represent individual patients. Three patients received a single dose of Pfizer vaccine prior to their two final samples.

In an overall time course, at three months five of the ten patients had lost detectable anti-RBD, a further two at four months and the remaining three at five months or more of follow up. Five patients were persistently sero-negative (33 samples) but had a positive-PCR result listed, two of these 33 unreactive samples displayed borderline binding ratios of between 0.8 and 0.9. Overall antibody reactivity persisted in recovery in 94 of 109 patients (86.2 %) (Fig. 7A). Only ten previously sero-reactive patients individuals lost detectable anti-RBD during follow-up (Fig. 7B). What is particularly noticeable is the strong antibody response to a single vaccine immunisation.

### 3.5 The first WHO international standard

**Fig. 8:**
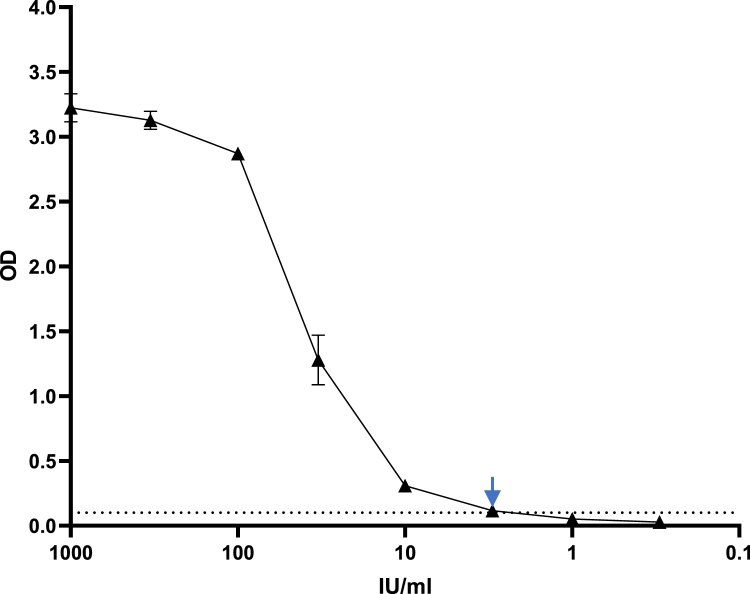
**Titration of First WHO International Standard.** Dotted line indicates the assay cut off, the arrow marks the sensitivity cut-off, 3 BAU/mL.

Serial dilution to extinction in normal human plasma of the first WHO International Standard for anti-SARS-CoV-2 immunoglobulin determined the sensitivity cut off of the Imperial Hybrid DABA to be 3 BAU/mL (Fig. 8, arrowed). The maximum threshold of the assay was determined to be 100 BAU/mL. Quantification of titres in excess of this required prior dilution in normal human plasma.

### 3.6 Relationship of anti-RBD level to disease status

**Fig. 9:**
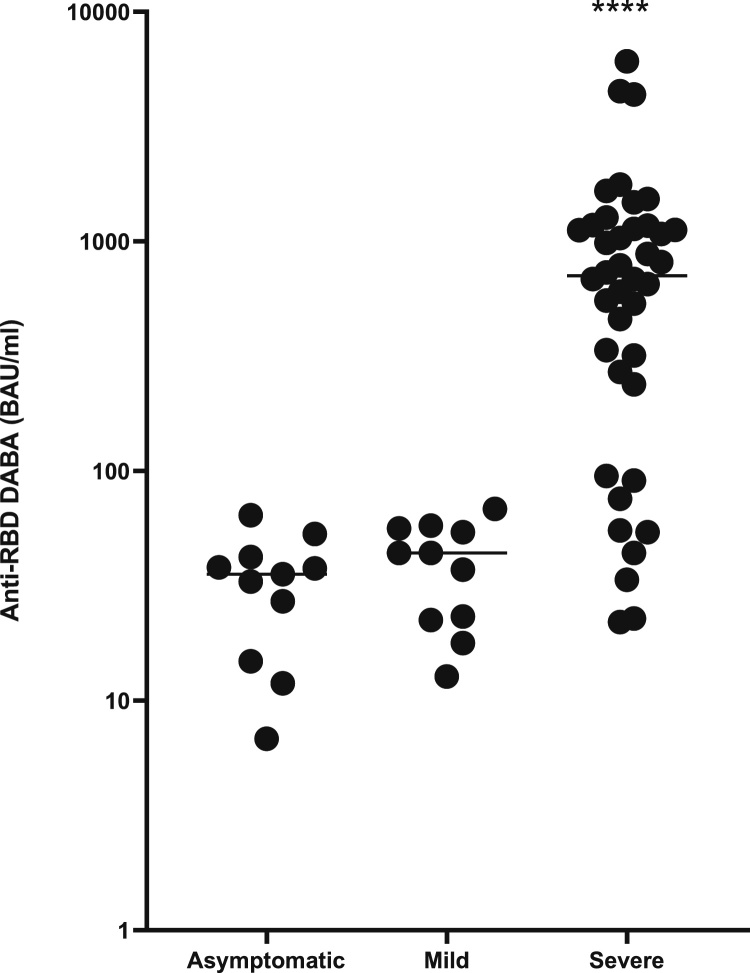
**Anti-RBD reactivity in three groups of samples defined by patient symptomatology.** The distribution of anti-RBD quantified by Hybrid DABA expressed in BAU/mL for three groups of patients with confirmed infections, asymptomatic, mild symptoms and those requiring hospitalisation. Average time post symptom onset (post diagnosis for asymptomatic) was 23, 36 and 28 days + respectively. **** p < 0.0001.

The anti-RBD BR was investigated in PCR-confirmed patients and hospital staff who had no symptoms (n = 10) and patients who were Covid-19 symptomatic at the time of their infection (n = 10). Convalescent patients admitted with Covid 19 (n = 40) from the ISARIC study provided the source for a third group. All samples were reactive and quantified in the DABA. The anti-RBD level was lowest in the asymptomatic patients (mean 33.2 BAU/mL), mildly elevated in the symptomatic patients (mean 39.8 BAU/mL) and significantly elevated in the patients hospitalised with Covid 19 (mean 1014.0 BAU/mL) **(**Fig. 9).

### 3.7 Quantitative antibody response in patients presenting with illness requiring hospitalisation

From the ISARIC4C study, 345 sera from 242 patients were available for anti-RBD quantification. Pre-dilution of the initial sample was needed for 68 % of samples which had BRs above 22 and all were ascribed an anti-RBD level in BAU/mL (Table 3). The mean level of anti-RBD varied in time, taking the day of recruitment as a series of convenience day 1 samples, and days 3 and 9 after recruitment as intermediates and convalescent. The most elevated titres were seen early in the admission, peaking at day 3 (mean 34,925 BAU/mL) falling at the nine-day sampling (mean 11,536 BAU/mL) and a further ten-fold by the day of convalescence sampling (mean 1042 BAU/mL).

**Table 3:** Quantitative analysis of hybrid DABA reactivity in three hundred and forty five ISARIC4C sera where timing of sampling was known at time of analysis.

**Patient source**	**Total**	**Seronegative (%)**	**Seropositive**	**Range of reactivity BAU/mL in reactive samples**	**Mean BAU/mL**	**Median BAU/mL**	**Standard Deviation BAU/mL**
Recruitment day 1	172	40 (23.3)	132	6-18,083	1,781	969	3,961
Day 3	98	15 (15.3)	83	11-1,144,444	34,925	1,439	124,803
Day 9	35	2 (5.7)	33	6-158,483	11,536	2,317	29,311
Convalescent day 28+	40	0 (0)	40	11-6,255	1,042	731	1,303

Samples found to be reactive in the hybrid DABA were ascribed a quantitative anti-RBD expressed as BAU/mL. Where necessary, each serum was pre-diluted in normal human plasma to a level where the reactivity fell onto the linear dilution curve.

### 3.8 Vaccine response in ferrets

Anti-RBD was detected in all ChadOx1 nCoV-19 immunised ferrets, but no reactivity was generated by ChadOx1 GFP immunisation (Table 4). At day 28, the mean reactivity was 53.1 BAU/mL and rose significantly on re-immunization to a mean of 261.1 BAU/mL at 35 days.

**Table 4:** Quantitative response to vaccine inoculation in Ferrets measured by hybrid DABA reactivity expressed in BAU/mL.

**Inoculum**	**Day**	**Number**	**Range**	**Mean**
**Vaccine ^$^**	28	11	9.7 – 110.3	53.1
	35	6	158.6 – 374.2	261.1
	42	6	41.7 – 147.2	100
**Placebo^∼^**	28	4	< 1	<1
	35	4	<1	<1
	42	4	<1	<1

Samples found to be reactive in the hybrid DABA were ascribed a quantitative anti-RBD expressed as BAU/mL. Where necessary, each serum was pre-diluted in normal human plasma to a level where the reactivity fell onto the linear dilution curve. ^$^ ChAdOx1nCoV-1^∼^ ChAdOx1 GFP.

### 3.9 Vaccine responses in humans

**Fig. 10:**
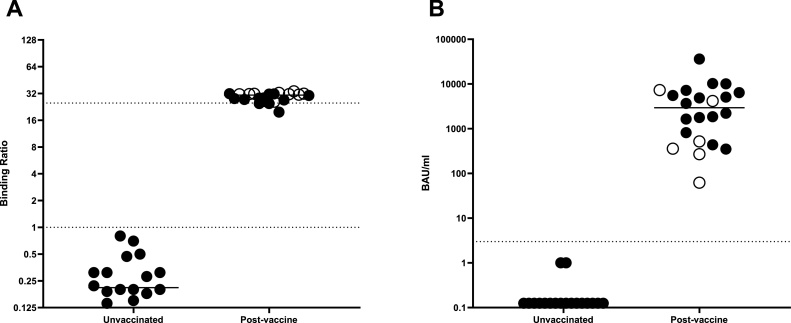
**Pre and post vaccine response in humans** Serological anti-RBD response to immunisation in BR (A) and WHO BAU/mL (B). Closed circles represent individuals that received Pfizer, and open circles those that received AstraZeneca. Dotted lines represent the quantitative limits of the assay. All post immunisation samples were taken at 14 days or longer following the second vaccine dose. P = 0.00049 (Wilcoxon matched pairs test).

In order to determine the utility of the hybrid DABA for detecting and measuring the vaccine responses in persons fully immunised samples were analysed from 28 persons who consented to giving samples for analysis. Where pre-immunisation samples were available the data are shown (Fig. 12). The vaccine response and the levels of anti-RBD generated are essentially above the limit of quantification when displayed as BRs (Fig. 10A). However, the vaccine response can also be ascribed international unitage (BAU/mL; Fig. 10B). All participants displayed a strong antibody response post vaccine, (mean: 5047 BAU/mL) although large range of responses was observed (66–36155 BAU/mL).

### 3.10 Discussion

The development and validation of accurate immunological assays are of the utmost importance both in gathering precise epidemiological data of the distribution of SARS-CoV-2 infections within a population and providing diagnostic support.

Many assays currently available (Deeks et al., 2020) for the detection of antibodies to SARS-CoV-2 have reported a very good performance, not necessarily confirmed by later studies. This is particularly so with anti-NP assays (Chew et al., 2020; Public Health England, 2020) on the Architect platform, in part because many of these assays were validated using samples obtained from hospital in-patients close in time to recovery. We present further evidence that patients with severe clinical presentation have higher antibody production than asymptomatic patients and those with mild disease (Chen et al., 2020; Okba et al., 2020; Hu et al., 2020) highlighting the importance of sensitive tests for large-scale population studies required to identify the common asymptomatic infections. Assays of low sensitivity risk underestimating the prevalence of SARS-CoV-2 infection, although corrections can be made for test performance (Diggle, 2011). More importantly, false reactivity may lead to dis-inhibition whereby individuals wrongly consider themselves refractory to reinfection and alter their behaviour.

Usually, in a DABA format the same proteins are used in the solid phase and as a detector in the fluid phase. However, in the Imperial Hybrid DABA, S1 comprises the solid phase and RBD conjugated to HRP is the revealing agent. This new and unique format of two different proteins acting in synergy contributes to the high specificity observed, since the only shared antigenicity in both preparations are the RBD epitopes. Importantly the assay, being species neutral, was able to detect and quantify the vaccine response in ferrets, predicting as we found that it is also able to detect and quantify anti-RBD in human vaccine recipients and may offer the possibility of more widespread zoonotic studies of animal seropositivity. The DABA format is preferentially sensitive to IgM due to the pentavalent nature of this antibody molecule. The apparent reduction of reactivity seen at day 42 in the ChadOx1 nCoV-19 immunised animals may reflect a shift to IgG maturation rather than a reduction of antibody *per se*. This may also explain the apparent short-term loss of vaccine response in humans.

A range of available assays have SARS-CoV-2 NP as a target. NP is more conserved between coronaviruses, potentially leading to problems with specificity (Yamaoka et al., 2020). Although the detection of anti-NP indicates prior infection and infers resistance to re infection, anti-RBD by definition will be neutralising and we observed a strong correlation between the Hybrid DABA BR and *in vitro* SARS-CoV-2 neutralisation IC_50_. The potential of the hybrid DABA in the identification of neutralising antibodies is important as vaccines are being rolled out and provides important antibody information to the individual, irrespective of whether naturally or vaccine acquired.

A rapid decline in antibody levels in the recovery phase (Ibarrondo et al., 2020; Bruni et al., 2020; Bölke et al., 2020; Wang et al., 2020; Yin et al., 2020) makes it difficult to predict the potential protection they may confer against re-infection; this may again be explained in part by IgG maturation. Our data suggest that specific anti-RBD remains stable (Fig. 7) and this may be for longer than other markers in short-term follow up. The high prevalence of samples strongly reactive for anti-RBD (Bölke et al., 2020) which are at or below the 1.4 BR cut off in the Abbott assay indicates a likely loss of detectable anti-NP in the Abbott assay following recovery though this does not exclude a *de novo* failure to generate anti-NP detectable in the Architect platform. Overall anti-NP was not detected in 511 of samples tested for both anti-NP and anti-RBD of which 294 (57.5 %) contained detectable anti-RBD. The rapid decay of antibody to SARS-CoV-2 is increasingly recognised for anti-NP detection on the Architect platform (Ibarrondo et al., 2020; Wang et al., 2020; Seow et al., 2020).

Although the long-term duration of the antibody response remains uncertain at this point, it is worth noting that a recent study by Lumley et al. (Lumley et al., 2020), demonstrates that antibody responses to CoVID in most people offered protection to reinfection for the ensuing six months. It may be that the DABA format is preferred for the detection of long standing seropositivity. In our experience the majority of persons recovering from SARS CoV-2 infection will have seroconverted by the time of recovery though a minority of five out a total of 109 remained seronegative throughout, in spite of a positive PCR result, and a small number of seropositive persons lose antibody on follow up. The reason for this is not known, however the briskness and magnitude of the response on vaccine challenge (Fig. 7B) is notable. The use of anti-RBD to measure vaccine response is advantageous as shown here with the hybrid DABA (Fig. 10). Using this format, the advantage of both high sensitivity and high specificity is considerable, identifying and quantifying a neutralising antibody response to immunisation with both current UK vaccines. The objective measurement of anti-RBD in the immunised person is of considerable utility at a time when the societal serological response to immunisation in humans is uncertain. The additional advantage of a species neutral assay of high sensitivity will also assist the research for zoonotic hosts of SARS CoV 2.

In the convenience samples from the ISARIC4C study the magnitude of the early response, probably in part due to the sensitivity of the class-neutral DABA to IgM was remarkable. This aggressive antibody response, though short-lived is certainly not trivial and the significance of this and its relatively swift effluxion remain obscure at this time.

Here we have validated an immunoassay that uses a new approach for the detection of total antibody of any class and species to SARS-CoV-2 which in effect measures anti-RBD with apparent absolute (100 %) specificity and exceptional (98.91 %) sensitivity. As seen in the ROC analysis the initial conservative generation of the cut-off may be reduced in future, using the mutant antigen, to give 99.6 % sensitivity whilst retaining exceptional specificity. The class neutral assay identified infection in the panel of 10 early convalescent asymptomatic infected individuals tested (Fig. 9) and has the additional attribute of species neutrality as demonstrated by the study of ferret samples, opening-up the potential for epi-zoological studies. Although this assay may be used in seroprevalence studies and as a confirmatory assay in combination with other serological tests including the under-performing Abbott SARS-CoV-2 anti-NP IgG assay its principal role at this time is to identify and measure the human response to immunisation with a range of vaccines and confer security by the detection of the generation of an antibody response in the immunised person indicating the generation of a virus neutralising response to the vaccine.

## Author statement

**Carolina Rosadas:** Writing - Original Draft, Supervision, Methodology, Validation, Formal Analysis, Investigation, Data Curation, Visualization.

**Maryam Khan:** Validation, Formal Analysis, Investigation, Data Curation.

**Eleanor Parker:** Methodology, Formal Analysis, Investigation, Data Curation, Writing - Review and Editing, Visualization, Supervision, Methodology.

**Federica Marchesin:** Validation, Formal Analysis, Investigation, Data Curation.

**Ksenia Katsanovskaja:** Validation, Investigation, Data Curation.

**Macià Sureda-Vives:** Validation, Investigation, Data Curation.

**Natalia Fernandez:** Validation, Investigation, Data Curation.

**Paul Randell:** Investigation, Resources.

**Ruth Harvey:** Investigation, Resources.

**Alice Lilley:** Investigation, Resources.

**Benjamin HL Harris:** Investigation, Resources.

**Mohamed Zuhair:** Investigation, Resources.

**Michael Fertleman:** Investigation, Resources.

**Samreen Ijaz:** Investigation, Resources.

**Steve Dicks:** Investigation, Resources.

**Charlotte-Eve Short:** Investigation, Resources.

**Rachael Quinlan:** Investigation, Resources.

**Graham P Taylor:** Investigation, Resources.

**Kai Hu:** Investigation, Resources.

**Paul McKay:** Investigation, Resources.

**Annachiara Rosa:** Investigation, Resources.

**Chloe Roustan:** Investigation, Resources.

**Mark Zuckerman:** Investigation, Resources.

**Kate El Bouzidi:** Investigation, Resources.

**Graham Cooke:** Investigation, Resources.

**Barnaby Flower:** Investigation, Resources.

**Maya Moshe:** Investigation, Resources.

**Paul Elliott:** Investigation, Resources.

**Alexandra J Spencer:** Investigation, Resources.

**Teresa Lambe:** Investigation, Resources.

**Sarah C Gilbert:** Investigation, Resources.

**Hugh Kingston:** Investigation, Resources.

**J Kenneth Baillie:** Investigation, Resources

**Peter JM Openshaw:** Investigation, Resources.

**Malcolm G Semple:** Investigation, Resources.

**ISARIC4C Investigators:** Investigation, Resources.

**Peter Cherepanov:** Investigation, Resources, Supervision, Conceptualization, Methodology.

**Myra O McClure:** Project Administration, Funding Acquisition, Writing - Original Draft, Writing - Review and Editing, Supervision, Methodology, Conceptualization.

**Richard S Tedder:** Project Administration, Funding Acquisition, Writing - Original Draft, Writing - Review and Editing, Supervision, Conceptualization, Methodology.

## Funding

This work is variously supported by grants from: the National Institute for Health Research (NIHR; awardCO-CIN-01), theMedical Research Council (MRC; grantMC_PC_19059 andMC_PC_19078), MRC NIHR (grantCV220-111) and by the NIHR Health Protection Research Unit (HPRU) in Emerging and Zoonotic Infections at University of Liverpool in partnership with Public Health England (PHE), in collaboration with Liverpool School of Tropical Medicine and the University of Oxford (award 200907), NIHR HPRU in Respiratory Infections at Imperial College London with PHE (award 200927), Wellcome Trust and Department for International Development (DID; 215091/Z/18/Z), the10.13039/100000865Bill and Melinda Gates Foundation (OPP1209135), Liverpool Experimental Cancer Medicine Centre (grant reference C18616/A25153), NIHR Biomedical Research Centre at Imperial College London (IS-BRC-1215-20013), EU Platform for European Preparedness Against (Re-) emerging Epidemics(PREPARE; FP7 project 602525), and NIHR Clinical Research Network for providing infrastructure support for this research. This work is supported by the Francis Crick Institute, which receives its core funding fromCancer Research UK (FC001061), the UK Medical Research Council (FC001061), and the10.13039/100004440Wellcome Trust (FC001061).

## Data sharing statement

Requests for data can be made at any time by contacting the corresponding author. The specific data and associated documents to be shared will be dependent on the nature of the individual request, and subject to the agreement of the principal investigator of the study from which the data is requested. All data provided will have patient identifying information excluded. ISARIC4C welcomes Data and Material access requests at https://isaric4c.net/sample_access/.

## Declaration of Competing Interest

The authors declare the following financial interests/personal relationships which may be considered as potential competing interests:

RST, MOM and PC report patent pending (Patent Application No. 2011047.4 for “SARS-CoV-2 antibody detection assay). All other authors declare no competing interests.

## Appendix A ISARIC4C Investigators

*Consortium Lead Investigator*: J Kenneth Baillie, *Chief Investigator:* Malcolm G Semple, *Co-Lead Investigator:* Peter JM Openshaw. *ISARIC Clinical Coordinator:* Gail Carson. *Co-Investigators*: Beatrice Alex, Benjamin Bach, Wendy S Barclay, Debby Bogaert, Meera Chand, Graham S Cooke, Annemarie B Docherty, Jake Dunning, Ana da Silva Filipe, Tom Fletcher, Christopher A Green, Ewen M Harrison, Julian A Hiscox, Antonia Ying Wai Ho, Peter W Horby, Samreen Ijaz, Saye Khoo, Paul Klenerman, Andrew Law, Wei Shen Lim, Alexander J Mentzer, Laura Merson, Alison M Meynert, Mahdad Noursadeghi, Shona C Moore, Massimo Palmarini, William A Paxton, Georgios Pollakis, Nicholas Price, Andrew Rambaut, David L Robertson, Clark D Russell, Vanessa Sancho-Shimizu, Janet T Scott, Thushan de Silva, Louise Sigfrid, Tom Solomon, Shiranee Sriskandan, David Stuart, Charlotte Summers, Richard S Tedder, Emma C Thomson, AA Roger Thompson, Ryan S Thwaites, Lance CW Turtle, Maria Zambon. *Project Managers*: Hayley Hardwick, Chloe Donohue, Ruth Lyons, Fiona Griffiths, Wilna Oosthuyzen. *Data Analysts*: Lisa Norman, Riinu Pius, Tom M Drake, Cameron J Fairfield, Stephen Knight, Kenneth A Mclean, Derek Murphy, Catherine A Shaw. *Data and Information System Managers*: Jo Dalton, James Lee, Daniel Plotkin, Michelle Girvan, Egle Saviciute, Stephanie Roberts, Janet Harrison, Laura Marsh, Marie Connor, Sophie Halpin, Clare Jackson, Carrol Gamble. *Data integration and presentation*: Gary Leeming, Andrew Law, Murray Wham, Sara Clohisey, Ross Hendry, James Scott-Brown. *Material Management:* William Greenhalf, Victoria Shaw, Sarah McDonald. *Patient engagement:* Seán Keating *Outbreak Laboratory Staff and Volunteers:* Katie A. Ahmed, Jane A Armstrong, Milton Ashworth, Innocent G Asiimwe, Siddharth Bakshi, Samantha L Barlow, Laura Booth, Benjamin Brennan, Katie Bullock, Benjamin WA Catterall, Jordan J Clark, Emily A Clarke, Sarah Cole, Louise Cooper, Helen Cox, Christopher Davis, Oslem Dincarslan, Chris Dunn, Philip Dyer, Angela Elliott, Anthony Evans, Lorna Finch, Lewis WS Fisher, Terry Foster, Isabel Garcia-Dorival, Willliam Greenhalf, Philip Gunning, Catherine Hartley, Antonia Ho, Rebecca L Jensen, Christopher B Jones, Trevor R Jones, Shadia Khandaker, Katharine King, Robyn T. Kiy, Chrysa Koukorava, Annette Lake, Suzannah Lant, Diane Latawiec, L Lavelle-Langham, Daniella Lefteri, Lauren Lett, Lucia A Livoti, Maria Mancini, Sarah McDonald, Laurence McEvoy, John McLauchlan, Soeren Metelmann, Nahida S Miah, Joanna Middleton, Joyce Mitchell, Shona C Moore, Ellen G Murphy, Rebekah Penrice-Randal, Jack Pilgrim, Tessa Prince, Will Reynolds, P. Matthew Ridley, Debby Sales, Victoria E Shaw, Rebecca K Shears, Benjamin Small, Krishanthi S Subramaniam, Agnieska Szemiel, Aislynn Taggart, Jolanta Tanianis-Hughes, Jordan Thomas, Erwan Trochu, Libby van Tonder, Eve Wilcock, J. Eunice Zhang. *Local Principal Investigators*: Kayode Adeniji, Daniel Agranoff, Ken Agwuh, Dhiraj Ail, Ana Alegria, Brian Angus, Abdul Ashish, Dougal Atkinson, Shahedal Bari, Gavin Barlow, Stella Barnass, Nicholas Barrett, Christopher Bassford, David Baxter, Michael Beadsworth, Jolanta Bernatoniene, John Berridge, Nicola Best, Pieter Bothma, David Brealey, Robin Brittain-Long, Naomi Bulteel, Tom Burden, Andrew Burtenshaw, Vikki Caruth, David Chadwick, Duncan Chambler, Nigel Chee, Jenny Child, Srikanth Chukkambotla, Tom Clark, Paul Collini, Catherine Cosgrove, Jason Cupitt, Maria-Teresa Cutino-Moguel, Paul Dark, Chris Dawson, Samir Dervisevic, Phil Donnison, Sam Douthwaite, Ingrid DuRand, Ahilanadan Dushianthan, Tristan Dyer, Cariad Evans, Chi Eziefula, Chrisopher Fegan, Adam Finn, Duncan Fullerton, Sanjeev Garg, Sanjeev Garg, Atul Garg, Effrossyni Gkrania-Klotsas, Jo Godden, Arthur Goldsmith, Clive Graham, Elaine Hardy, Stuart Hartshorn, Daniel Harvey, Peter Havalda, Daniel B Hawcutt, Maria Hobrok, Luke Hodgson, Anil Hormis, Michael Jacobs, Susan Jain, Paul Jennings, Agilan Kaliappan, Vidya Kasipandian, Stephen Kegg, Michael Kelsey, Jason Kendall, Caroline Kerrison, Ian Kerslake, Oliver Koch, Gouri Koduri, George Koshy, Shondipon Laha, Steven Laird, Susan Larkin, Tamas Leiner, Patrick Lillie, James Limb, Vanessa Linnett, Jeff Little, Michael MacMahon, Emily MacNaughton, Ravish Mankregod, Huw Masson, Elijah Matovu, Katherine McCullough, Ruth McEwen, Manjula Meda, Gary Mills, Jane Minton, Mariyam Mirfenderesky, Kavya Mohandas, Quen Mok, James Moon, Elinoor Moore, Patrick Morgan, Craig Morris, Katherine Mortimore, Samuel Moses, Mbiye Mpenge, Rohinton Mulla, Michael Murphy, Megan Nagel, Thapas Nagarajan, Mark Nelson, Igor Otahal, Mark Pais, Selva Panchatsharam, Hassan Paraiso, Brij Patel, Natalie Pattison, Justin Pepperell, Mark Peters, Mandeep Phull, Stefania Pintus, Jagtur Singh Pooni, Frank Post, David Price, Rachel Prout, Nikolas Rae, Henrik Reschreiter, Tim Reynolds, Neil Richardson, Mark Roberts, Devender Roberts, Alistair Rose, Guy Rousseau, Brendan Ryan, Taranprit Saluja, Aarti Shah, Prad Shanmuga, Anil Sharma, Anna Shawcross, Jeremy Sizer, Manu Shankar-Hari, Richard Smith, Catherine Snelson, Nick Spittle, Nikki Staines, Tom Stambach, Richard Stewart, Pradeep Subudhi, Tamas Szakmany, Kate Tatham, Jo Thomas, Chris Thompson, Robert Thompson, Ascanio Tridente, Darell Tupper-Carey, Mary Twagira, Andrew Ustianowski, Nick Vallotton, Lisa Vincent-Smith, Shico Visuvanathan, Alan Vuylsteke, Sam Waddy, Rachel Wake, Andrew Walden, Ingeborg Welters, Tony Whitehouse, Paul Whittaker, Ashley Whittington, Meme Wijesinghe, Martin Williams, Lawrence Wilson, Sarah Wilson, Stephen Winchester, Martin Wiselka, Adam Wolverson, Daniel G Wooton, Andrew Workman, Bryan Yates, and Peter Young.
